# Fabrication of Paper Sheets Coatings Based on Chitosan/Bacterial Nanocellulose/ZnO with Enhanced Antibacterial and Mechanical Properties

**DOI:** 10.3390/ijms22147383

**Published:** 2021-07-09

**Authors:** Joanna Jabłońska, Magdalena Onyszko, Maciej Konopacki, Adrian Augustyniak, Rafał Rakoczy, Ewa Mijowska

**Affiliations:** 1 Department of Chemical and Process Engineering, Faculty of Chemical Technology and Engineering, West Pomeranian University of Technology in Szczecin, Piastow Ave. 42, 71-065 Szczecin, Poland; maciej.konopacki@zut.edu.pl (M.K.); adrian.augustyniak@zut.edu.pl (A.A.); rafal.rakoczy@zut.edu.pl (R.R.); 2 Department of Nanomaterials Physicochemistry, Faculty of Chemical Technology and Engineering, West Pomeranian University of Technology in Szczecin, Piastow Ave. 49, 71-065 Szczecin, Poland; magdalena.onyszko@zut.edu.pl (M.O.); ewa.borowiak-palen@zut.edu.pl (E.M.); 3 Chair of Building Materials and Construction Chemistry, Technische Universität Berlin, Gustav-Meyer-Allee 25, 13355 Berlin, Germany

**Keywords:** biopolymers, paper packaging, antimicrobial activity, nanoparticles

## Abstract

Here, we designed paper sheets coated with chitosan, bacterial cellulose (nanofibers), and ZnO with boosted antibacterial and mechanical activity. We investigated the compositions, with ZnO exhibiting two different sizes/shapes: (1) rods and (2) irregular sphere-like particles. The proposed processing of bacterial cellulose resulted in the formation of nanofibers. Antimicrobial behavior was tested using *E. coli* ATCC^®^ 25922™ following the ASTM E2149-13a standard. The mechanical properties of the paper sheets were measured by comparing tearing resistance, tensile strength, and bursting strength according to the ISO 5270 standard. The results showed an increased antibacterial response (assigned to the combination of chitosan and ZnO, independent of its shape and size) and boosted mechanical properties. Therefore, the proposed composition is an interesting multifunctional mixture for coatings in food packaging applications.

## 1. Introduction

The food packaging industry is a source of tons of plastic waste every year. It raises environmental concerns about greenhouse gas emissions, a growing carbon footprint, and the welfare of sea animals and birds [1,2,3,4]. Therefore, attempts to use more eco-friendly materials, such as biopolymers, are intensified. Those materials can exhibit good properties when incorporated into the mass of cellulose used for paper production or can be applied as coatings of the paper sheets [5,6].

One of the biopolymers used in packaging applications is chitosan—a polysaccharide that consists of d-glucosamine and n-acetyl-d-glucosamine monomers. It is obtained as a result of deacetylation of chitin—an ingredient of crustacean shells. Chitosan has gained much recognition due to its properties such as biodegradability, non-toxicity, filmmaking, and antimicrobial potential [7]. 

Bacterial cellulose is a biopolymer obtained from stationary or dynamic bacterial cultures. One of the most recognized producers belongs to the *Komagataeibacter* genus (previously called *Acetobacter xylinus* or *Gluconacetobacter xylinus*) [8]. Bacterial cellulose is created at the edge of the liquid–gas phases in the form of hydrogel [9]. It is characterized by significant water retention, the possibility of obtaining different shapes, and good mechanical properties compared to plant cellulose due to the lack of lignin or hemicellulose in its structure. Moreover, the crystallinity of bacterial cellulose is higher than in plant cellulose, resulting in good mechanical properties [7]. 

One of the most promising application areas of bacterial cellulose is the paper and textile industry, where it can be used as a reinforcing agent in the form of nanocrystals (BCNC) or nanofibers (BCNF) [10]. Bacterial cellulose nanocrystals, also called nanowhiskers, are characterized by a diameter of 4–25 nm and a length of 100–1000 nm and occur in the shape of needles. They can also be obtained in the form of nanofibers, which have a length of around 10–100 nm and are obtained by physical processes such as homogenization, ball milling, or sonication [11,12]. 

Moreover, the possibility of incorporating various nanoparticles into different biopolymers provides new opportunities for their application. In addition, nanoparticles, such as silver, copper, or zinc oxide, are used as one of the components to enhance the antimicrobial properties of paper packaging. Zinc oxide is widely used in research concerning electronics, photocatalysis, sensors, construction materials, and medicine [13,14]. It is considered GRAS (generally recognized as safe) by the U.S. Food and Drug Administration (FDA) and can be used as a part of food packaging [15,16]. Zinc oxide exhibits an efficient antimicrobial response and is mainly incorporated into the polymer matrix as an additive. 

Many studies have utilized the above-mentioned components in the fabrication of films or paper coatings. Salari et al. [7] used BCNC, chitosan, and silver nanoparticles to fabricate nanocomposites in the form of film. It expressed good barrier and mechanical properties and exhibited antimicrobial activity. George and Siddaramaiah [17] formulated a composite based on BCNC and gelatin, which resulted in the enhancement of mechanical properties compared to pristine gelatin film. Moreover, George et al. [18] noted higher thermal stability and better mechanical properties of BCNC-poly(vinyl alcohol) composite in comparison to the film from the pure polymer. In turn, Viana et al. [19] obtained films based on pectin and bacterial cellulose in the form of BCNF. The films were characterized with better mechanical properties and water resistance than pristine pectin films. Yadav et al. [20] produced chitosan-based films containing ZnO nanoparticles and gallic acid that exhibited good antimicrobial and mechanical properties and antioxidant behavior. Some authors prepared coated paper samples. Divsalar et al. [21] prepared a paper coated with chitosan, ZnO, and nisin that showed antibacterial activity. Prasad et al. [22] obtained papers with ZnO and soluble starch. The samples exhibited improved whiteness, oil absorbency, and excellent antifungal and UV-protecting properties. 

To our knowledge, only one research article covered the use of chitosan, bacterial cellulose, and ZnO [23]. However, the application and processing of the components differed from the method applied in this paper. The authors prepared antibacterial dressing by immersion of oxidized bacterial cellulose hydrogels in chitosan solution and thereafter, synthesized ZnO particles on the surface. Here, we focus on the functionalization of the paper sheets to provide good antibacterial and mechanical properties; therefore, two well-known antibacterial components, i.e., chitosan and ZnO particles, were employed. Bacterial cellulose was applied in the form of nanofibers to enhance mechanical properties. In addition, chitosan is characterized by excellent film-making properties and was applied as the matrix in which the ZnO-modified bacterial cellulose was incorporated. The proposed method of fabrication significantly reduces the impact on the environment. The first example is the use of biopolymers as an alternative to non-degradable ingredients. Secondly, the concentration of ZnO particles is relatively low to avoid a possible cytotoxic effect, while the antibacterial potential of the composites is maintained by the use of chitosan. Moreover, bacterial cellulose processing is based on ball milling and sonication instead of acid hydrolysis, which requires the use of concentrated HCl or H_2_SO_4_. Direct synthesis of ZnO on the surface of bacterial cellulose fibers reduces the number of performed procedures and time of experiments.

Here, we focused on the fabrication of paper coating based on chitosan, bacterial cellulose, and ZnO to induce antimicrobial response and enhance mechanical properties with respect to pristine paper. The processing of bacterial cellulose sheets was performed via lyophilization, ball milling, sonication, and re-lyophilization. It resulted in the formation of bacterial cellulose nanofibers (BCNF). These nanostructures were later decorated with ZnO particles in two different shapes: rods and irregular sphere-like particles. Therefore, the morphology and their performance when used in paper coatings were efficiently tuned.

## 2. Results

### 2.1. Characterization of the Composites and Coatings

**Figure 1:**
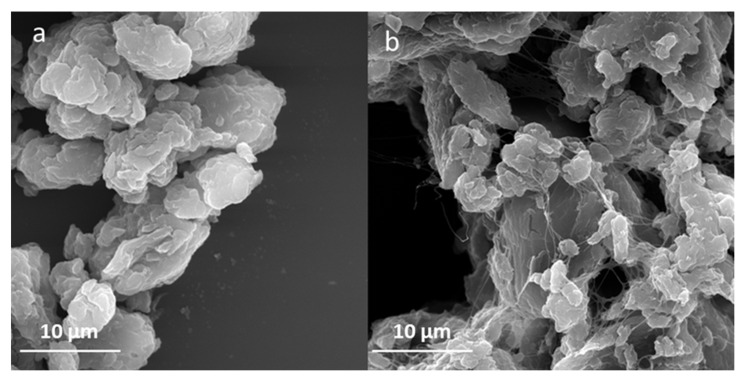
SEM micrographs of bacterial cellulose (**a**) before and (**b**) after sonication.

**Figure 2:**
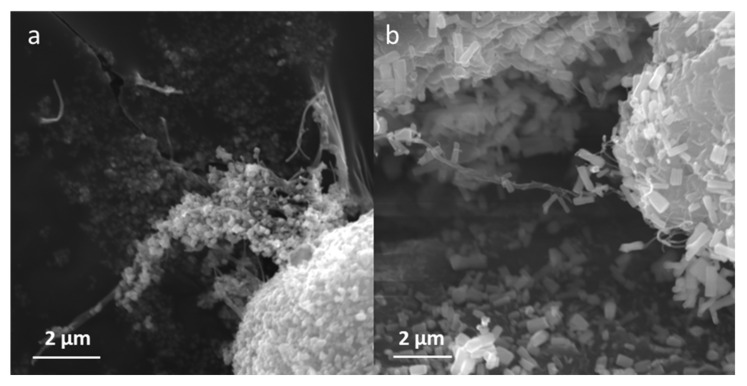
SEM micrographs of bacterial cellulose modified with ZnO (**a**) at room temperature; (**b**) at 80 °C.

SEM analysis allowed the change in bacterial cellulose structure to be observed after 1-hour sonication. The process resulted in partial separation of the nanofibers. The comparison of non-sonicated, agglomerated bacterial cellulose and the sonicated sample is presented in Figure 1. Moreover, SEM enabled the preliminary verification of ZnO synthesis on the bacterial cellulose surface. The micrographs in Figure 2 present the modification of morphology of the nanocomposites affected by different temperatures applied during metal oxide deposition: room temperature (a) and 80 °C (b). Noticeable differences were observed, such as the size and the shape of the ZnO particles. ZnO synthesized at room temperature is characterized by nanometric size and non-uniform structure. Moreover, the particles were agglomerated. However, the shape of ZnO synthesized at 80 °C can be described as elongated rods with a diameter ~200 nm and length 1 µm. 

**Figure 3:**
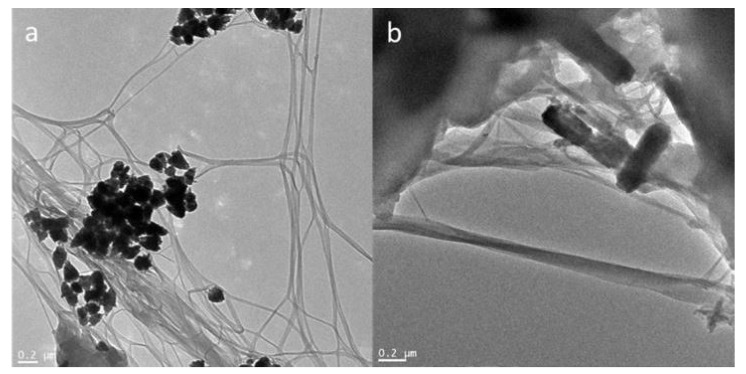
TEM micrographs of bacterial cellulose-ZnO composites (**a**) BCsonTZnO; (**b**) BCson80ZnO.

**Figure 4:**
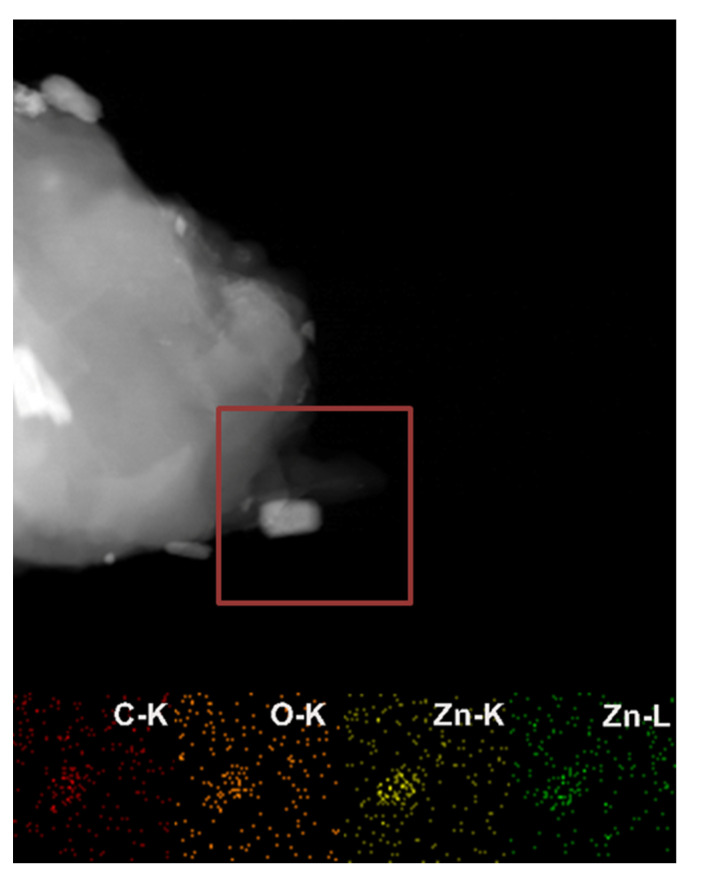
Elemental analysis of BCson80ZnO composite (the red square indicates the analyzed region of the sample).

TEM analysis allowed the successful fabrication of the bacterial cellulose-ZnO composites to be confirmed and detailed analysis of their morphology. Figure 3 presents images of all the obtained composites. The BCsonTZnO composite consisted of agglomerated ZnO particles in the network of relatively well-separated nanofibers of bacterial cellulose. On the other hand, the BCson80ZnO composite contained long ZnO rods placed on the fibers. TEM analysis confirmed that sonication induced the fabrication of bacterial cellulose nanofibers. Moreover, elemental mapping of the obtained composites was carried out to assess the location of the elements in the samples. This confirmed the presence of carbon (C-K), oxygen (O-K), and zinc (Zn-K and Zn-L) (Figure 4). 

**Figure 5:**
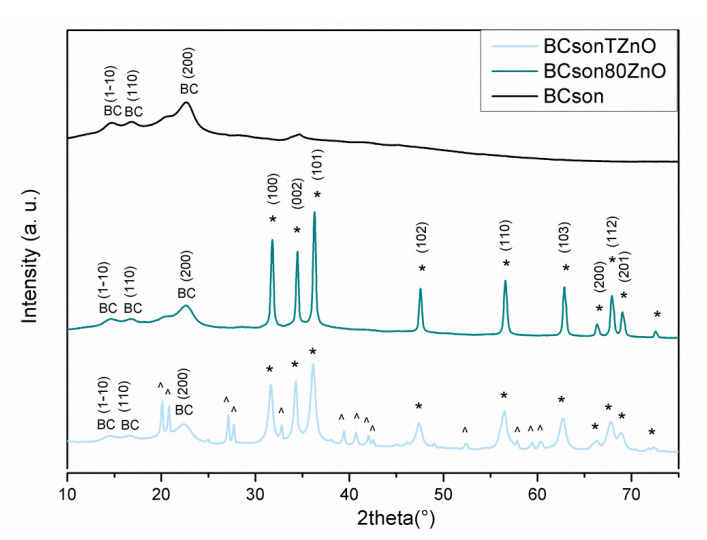
XRD analyses of the obtained bacterial cellulose-ZnO. * ZnO, BC—bacterial cellulose, ^ Zn(OH)_2_ (a.u.–arbitrary unit).

Figure 5 presents the X-ray diffraction patterns taken for the bacterial cellulose composites. The patterns revealed that the obtained samples exhibit a typical crystalline form of cellulose I. For each sample, major diffraction peaks at 14.6°, 16.7°, and 22.6° corresponding to the crystallographic planes of (110), (110), and (200), respectively, could be recognized. However, several additional peaks in all nanocomposite samples can be observed. Sharp and intense Bragg reflections are positioned at 31.05°, 34.58°, 36.48°, 47.68°, 56.78°, 63.07°, 66.53°, 68.11°, and 69.29°. These reflections are well-matched with the usually reported signals of ZnO wurtzite hexagonal structure attributed to (100), (002), (101), (102), (110), (103), (200), (112), and (201), respectively (following standard PDF card no. 01-079-0207). The obtained XRD pattern of BCsonTZnO displays a few additional diffraction peaks in the range of 20–65°. These comparatively weak signals are assigned to zinc hydroxide, which is an intermediate product during the formation of ZnO in alkaline solution. Zn(OH)_2_ can only be found in the sample prepared at room temperature. 

**Figure 6:**
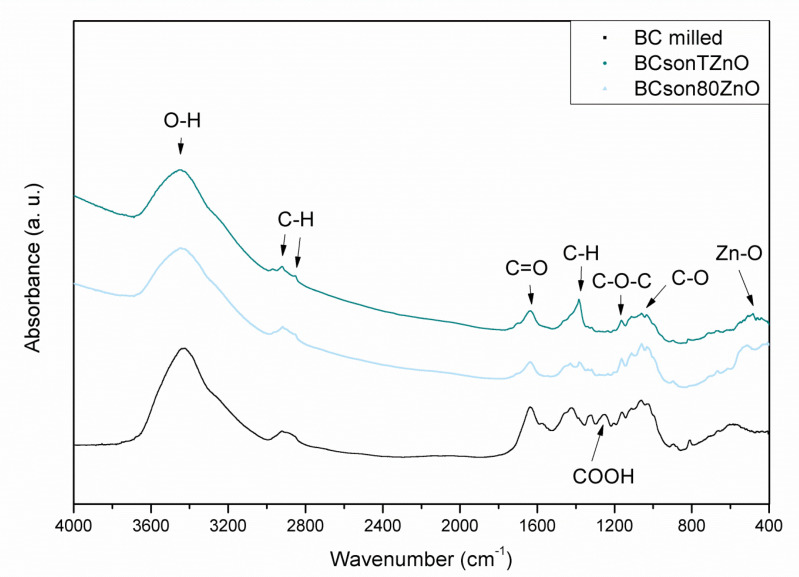
FT-IR analyses of the obtained bacterial cellulose-ZnO composites (a.u.–arbitrary unit).

Figure 6 shows FT-IR spectra of two representatives of bacterial cellulose in respect to the pristine bacterial cellulose sample. All spectra contain the main bands: at 3432 cm^−1^ attributed to the O-H stretching, 2921 cm^−1^ and 2853 cm^−1^ characteristic of C-H stretching of the CH2 and CH3 groups, 1165 cm^−1^ assigned to C–O–C antisymmetric bridge stretching of 1,4-β-d-glucoside and 1053 cm^−1^ corresponding to bending of the C–O–H bond of carbohydrate. The band centered around 1626 cm^−1^ is due to the O-H bending of adsorbed water. It is present in all samples, and its intensity is the highest for the pure BC sample, indicating the hydrophilic character of the material. There are some differences between BC and all obtained composites. Subtle changes can be observed in the low-wavenumber region, where the bands characteristic of Zn-containing groups have been observed (400–500 cm^−1^). The results may be related to the strong chemical interaction between cellulose and ZnO phases. Spectra obtained for the composites show broadening of the bands in the region 3200–3600 cm^−1^, probably due to the rearrangement and increase in the hydroxyl group content. Moreover, the signals in the 2850–2950 cm^−1^ region have become more distinctive but less intense after the functionalization process. 

### 2.2. Antibacterial Properties

**Figure 7:**
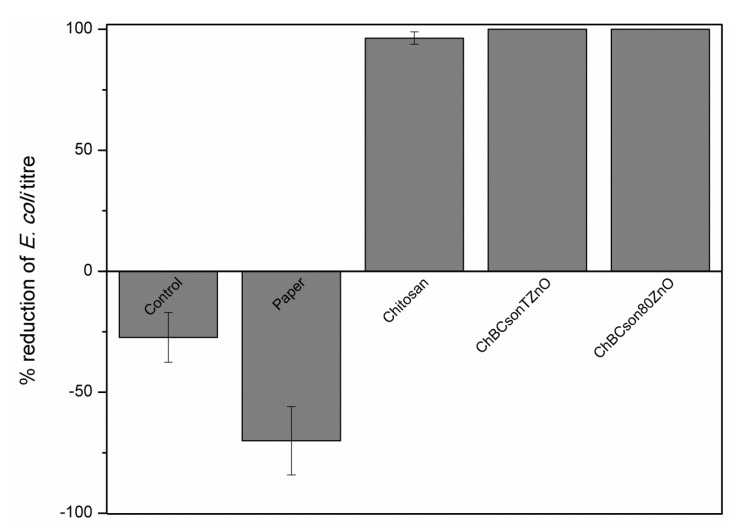
Antibacterial properties assay according to ASTM E2149-13a standard (chitosan—paper coated with chitosan, ChBCsonTZnO—paper coated with chitosan, sonicated bacterial cellulose and ZnO synthesized at the room temperature, ChBCson80ZnO—paper coated with chitosan with sonicated bacterial cellulose and ZnO synthesized at 80 °C).

The results for antibacterial properties are presented in Figure 7. The antimicrobial assay showed a 96.36% reduction in *E. coli* titer after incubation with chitosan-coated paper and a 100% reduction with both papers coated with fabricated chitosan-bacterial cellulose-ZnO composites. In the case of pristine paper, the titer of bacteria was increased by 70%. In this study, it is crucial to point out that there is no shape/size effect of ZnO on antimicrobial response.

### 2.3. Mechanical Properties

To verify the effect of the various coatings on the mechanical properties of the non-coated and coated paper, sheets were subjected to mechanical tests. The tests evaluated the tensile strength, tearing strength, and bursting strength. For each test, eight different paper sheet samples were measured and the average values were calculated. The standard deviation of measured values was also indicated. Because of the slight changes in weight of the coated paper sheets, all the collected data are presented as a strength index, which is a preferable factor to compare the samples with different grammages. Table 1 summarizes the obtained results, indicating enhancement of mechanical parameters of the coated samples in respect to the pristine paper. The tensile strength value of the coated paper sheets was increased by (on average) ~8.5% as compared to the reference sample. Tearing strength value was improved (on average) by 14.5% in the machine direction and even more in the cross-machine direction—18%. The bursting strength value of the coated paper sheets also increased by 16.5% on average. Interestingly, both composite-coated samples have shown similar mechanical performance concerning tear index, indicating that the shape of ZnO did not affect it. However, tensile strength and burst index differed in the samples and sample ChBCson80ZnO exhibited better mechanical properties than sample ChBCsonTZnO. The tensile index shows that both samples containing bacterial cellulose and ZnO exhibited higher values than pristine chitosan and pristine paper. It is also worth underlining that paper sheets coated with pristine chitosan presented the best tear index (CD) value from all of the analyzed samples. Conducted research confirms that all coated samples had better mechanical properties than uncoated paper, which can be mostly assigned to chitosan. Only the tensile index was significantly improved in the case of composites (in comparison with chitosan coating and non-coated paper). 

## 3. Discussion

Our study indicated that fabricated paper coated with composites based on chitosan, bacterial cellulose, and ZnO exhibited enhanced antibacterial activity and mechanical performance. The antibacterial effect may be explained by the analysis of the antimicrobial properties of chitosan and ZnO. In the case of chitosan, its properties are associated with many factors, e.g., pH of the solution, molecular weight and degree of acetylation, or the surface of contact with the cells [24,25]. There are several mechanisms of the antimicrobial activity of chitosan described in the literature [26]: (1) The first one is explained as an electrostatic attraction of positively charged chitosan and negatively charged components on the surface of bacterial cells. (2) Another proposed mechanism is based on the ability of chitosan to penetrate the cell, bind with DNA and disturb the transcription and translation processes. An effect of such action is the disorganization of protein synthesis crucial for sustaining cells’ homeostasis. The last (3) mechanism relies on the chelation of metal ions by amino groups of chitosan. Such effects are observed in pH above six, since amino groups become unprotonated and the pair of electrons are donated to metal. This results in the creation of metal complexes. 

The bactericidal properties of zinc oxide were described by many authors and are a well-known phenomenon [27,28]. The antimicrobial activity of zinc oxide nanoparticles is hypothesized to be based on three main mechanisms: (1) the release of Zn^2+^ ions, (2) interaction of nanoparticles with bacteria, which may result in cell disruption, (3) production of reactive oxygen species (ROS) [29,30]. In the presented research, the size/shape of the particles was assessed by transmission electron microscopy. The size of ZnO particles was estimated as nanometric in the case of ZnO synthesized at room temperature, while ZnO synthesized at 80 °C was characterized by micrometric dimensions. On that basis, we hypothesize that the most probable antimicrobial mechanism of the fabricated coatings was acting through zinc ions released to the buffer used in the conducted assay. Such a process is connected to the amphoteric nature of ZnO in water solutions. The ions bind with the thiol groups of the enzymes involved in cell respiration, which disorganizes their functions and leads to cell death [29,30]. Therefore, ZnO particles are frequently used in the fabrication of antimicrobial films or paper. However, the cytotoxic effect of high concentrations of ZnO particles was frequently reported in the literature [31,32]. Therefore, we decided to apply a relatively small amount of ZnO in the prepared composites by employing another ingredient—chitosan. Chitosan is also known as an antimicrobial agent and is recognized as a non-toxic, biodegradable polysaccharide. It is the second most abundant natural polymer, after plant cellulose [33]. Moreover, it exhibits excellent coating properties and therefore, it played a dual role in the prepared composites—the film-forming matrix in which ZnO-modified bacterial cellulose is incorporated and the antimicrobial agent. However, the antimicrobial activity of chitosan is limited and depends on many factors [7,34,35]. Therefore, two antimicrobial factors were chosen to fabricate the paper coating to obtain the additive effect. Other published papers employ the same strategy, i.e., the use of two antimicrobial agents [23,36]. The assay of antimicrobial activity of the composites revealed that the combination of chitosan and ZnO was more effective than chitosan alone and led to a 100% reduction in bacterial titer, while paper coated with pristine chitosan exhibited a 96.36% reduction.

The benefits of using nanocellulose in the improvement of mechanical properties have already been addressed in the literature [37]. The addition of cellulose nanostructures having a high surface-to-volume ratio improves the formation of hydrogen bonding within cellulose pulp, which increases density, boosting the mechanical performance of paper. Tanpichai et al. [38] reported a significant enhancement of mechanical properties of paper composed of 50 wt% nanofibrillated cellulose (NFC). Tensile strength and strain were 10-fold and 3-fold higher than those of the paper without NFC, respectively. Jin and his coworkers [39] have designed paper coating using nanofibrillated cellulose as a coating agent. The results of their studies revealed that increased NFC addition led to the enhancement of tensile strength, which was achieved as a function of the NFC addition in the paper coating system. They demonstrated that 0.03% addition of NFC improved the tensile strength of coated paper by approximately 2.5%.

Recent studies have also found the viability of chitosan as a paper coating not only for the implementation of antibacterial features but also to improve its mechanical properties. This enhancement of mechanical properties might be due to the fact that pores within the paper can be filled with polymer molecules. Zakaria et al. [40] have coated A4 paper with 2 wt% of chitosan solution and reported some changes in paper strength and toughness in comparison to uncoated samples. The burst and tensile strength values for the coated paper have increased by 9% and 6%, respectively. Moreover, it has been reported that chitosan adheres well to the fiber surfaces, causing the formation of bridges between inter-fiber distances [41]. Thus, coating paper with a mixture of chitosan and nanocellulose can benefit from the formation of bonds between cellulose fibers and nanocellulose fibrils. Therefore, exploiting the additive effect of bacterial cellulose nanofibers and chitosan for the reinforcement of mechanical properties is possible. 

It is worth underlining that, in our studies, the improvement of mechanical properties of the coated paper sheets can mostly be assigned to chitosan. Overall, the ChBCson80ZnO sample exhibited the best mechanical properties, excluding tear index (CD).

## 4. Materials and Methods

Zinc acetate dihydrate (Zn(CH_3_COO)_2_ × 2H_2_O), acetic acid, and medium molecular weight chitosan (190,000–310,000 Da, 75–85% deacetylated) were purchased from MiliporeSigma (Darmstadt, Germany). Sodium hydroxide (NaOH) was supplied by Chempur (Piekary Śląskie, Poland).

### 4.1. Bacterial Cellulose Growth, Processing and Functionalization with ZnO

Bacterial cellulose was produced by *Komagataeibacter xylinus* ATCC^®^ 53524™. The culture was conducted for seven days at 30 °C in S&H 1717 ATCC medium (bactopeptone 5 g/L, yeast extract 5 g/L, Na_2_HPO_4_ 2.7 g/L, citric acid 1 hydrate 1.15 g/L). After sterilization, the aqueous solution of mannitol (20% *w*/*w*) was added at the amount of 20 g/L, and the medium was buffered to pH = 5. Based on the literature [42], mannitol is one of the best carbon sources for selected bacteria strains—it results in high BC yield in a short time and the highest crystallinity index. Next, the inoculum (20 mL) was transferred to the new portion of the sterile medium and cultivated in a stationary culture (1.2 L) on polypropylene trays for the next eight days at 30 °C. After the incubation time, the sheet of bacterial cellulose was harvested, rinsed thoroughly with distilled water, and incubated in 0.1 M NaOH solution at 80 °C for 30 min to remove the residual cells. After that, the sheet was rinsed again with distilled water to remove the residue of NaOH. 

Bacterial cellulose processing was based on the lyophilization of the whole sheet, ball milling (1 h), sonication for 1 h, and re-lyophilization. This approach resulted in the formation of bacterial cellulose nanofibers (BCNF) due to mechanical processing. 

The synthesis of the zinc oxide particles was conducted directly on bacterial cellulose samples according to the modified method by Ali et al. [43]. The calculated ratio of the bacterial cellulose:ZnO was 1:2. Briefly, 750 mg of the bacterial cellulose was inserted into 250 mL of distilled water and stirred with a magnetic stirrer (300 rpm, 30 min) to obtain homogenous dispersion. Next, Zn(CH_3_COO)_2_ × 2H_2_O was added to the flask and stirred for the next 0.5 h. After that, 0.1M NaOH was introduced using a vacuum pump with the flow 3 mL/minute (Programmable Microfluidics Syringe Pump NE-1002X, Ala Scientific, Farmingdale, NY, USA). After this step, the obtained pellet was washed with ethanol, and the residues were rinsed with distilled water and filtered on a Whatman filter (0.2 µm) until pH = 7 was reached. Then, the samples were dried at 60 °C.

### 4.2. Preparation of Chitosan Composites

The chosen concentration of the chitosan solution was 1.5% *w*/*v* in 1% *v*/*v* water solution of the acetic acid based on preliminary attempts to coat a sheet of paper. The solution was placed on the magnetic stirrer (300 rpm) and sonicated for 15 min until a homogeneous suspension was obtained. The final composites were prepared by adding 10% *w*/*w* (in relation to the dry mass of chitosan) of bacterial cellulose modified with ZnO into the chitosan solution. The composites were stirred with a magnetic stirrer for 30 min and sonicated (15 min) to obtain homogenous dispersion of the modified bacterial cellulose particles. The final concentrations of bacterial cellulose and chitosan equaled 6.77% and 3.33%, respectively. Coating of the kraft paper sheets (grammage 90 g/m^2^, Arctic Paper S.A., Poznan, Poland) was conducted with a coating machine (RK K Control Coater, Royston, UK) by distribution of the nanocomposite on the surface of the paper sheet with a squeegee with a coating thickness of 12 µm. Afterward, the coated papers were air-dried. All the samples are listed in Table 2.

### 4.3. Characterization

SEM analysis was conducted to assess the morphology of the bacterial cellulose and obtained ZnO particles. The samples were prepared on carbon tape and sputtered with chromium. The voltage of the electron beam equaled 20 kV (VEGA3, TESCAN, Brno, Czech Republic). TEM analysis was carried out to precisely assess the size of the obtained ZnO particles and the efficiency of the sonication of the bacterial cellulose. The samples were sonicated and placed on a copper grid and incubated at 60 °C to remove the water. The electron beam voltage used was 200 kV (Tecnai F30, Thermo Fisher Scientific, Waltham, MA, USA). Additional elemental analysis was run to identify the elements of the composites. XRD allowed the confirmation of the presence of the bacterial cellulose and ZnO particles in the samples before insertion into the chitosan solution. The powdered sample was placed on the rack, and the measurements were conducted in the range of 10–75°, with the copper lamp as a radiation source (Kα1 = 1.54056 Å) (Aeris Research, Malvern Panalytical, Malvern, UK). The FT-IR method (Nicolet 6700 FT-IR, Thermo Fisher Scientific, Waltham, MA, USA) was used to identify the characteristic groups of the used components. The samples were prepared by forming tablets with KBr. The measurements were conducted in the range of 4000–400 cm^−1^.

### 4.4. Antibacterial Properties

The assessment of the antimicrobial properties of the coated paper was conducted according to ASTM E2149-13a standard (Standard Test Method for Determining the Antimicrobial Activity of Antimicrobial Agents Under Dynamic Contact Conditions). The bacteria were kept at −20 °C in a trypticase soy broth (TSB) medium containing 10% glycerol. Before the study, the microorganisms were revived on trypticase soy agar (TSA) and incubated at 37 °C for 24 h. For experiments, bacteria were inoculated into falcon-type tubes (50 mL) containing 30 mL of TSB and incubated overnight at 37 °C on a rotary shaker for 18 h. Then, the cultures were diluted in phosphate buffer (0.3 mM KH_2_PO_4_), and the obtained inoculum was used for the experiments. One gram of each paper sample was weighed on the analytical balance and inserted into sterile Erlenmeyer flasks. Next, 50 mL of the inoculum was added, the solution was mixed by hand, and 100 µL of each sample was collected for the plating. All the samples were incubated at room temperature for 1 h with shaking (150 rpm). Afterward, 100 µL of the cultures were collected for plating on plate count agar (PCA) in two repetitions. The plates were incubated at 37 °C for 24 h. After the incubation, the colonies were counted, and the reduction percentage was calculated in comparison to the control.

### 4.5. Mechanical Properties

The mechanical properties of the functionalized and non-functionalized paper samples were investigated. For comparative purposes, non-coated paper sheets were also tested and used as a reference sample. The mechanical tests were carried out at the Arctic Paper company in an air-conditioned room (23 °C, 50% of the humidity level). Tearing resistance was determined using an Elmendorf apparatus (Lorentzen & Wettre, Zurich, Switzerland) according to ISO 1924-2. The width and length of the tested paper strips were 15 and 100 mm, accordingly. The tensile strength measurements were conducted on the automatic tensile tester (Messmer Büchel, K465, Veenendaal, The Netherlands), according to ISO 1974 on samples consisting of 4 paper sheets. The bursting strength was tested on the bursting strength tester (Messmer Büchel, Veenendaal, The Netherlands) according to ISO 2758 on paper samples with dimensions of more than 70 mm × 70 mm.

## 5. Conclusions

In summary, paper sheets coated with composites based on chitosan, bacterial cellulose (nanofibers), and ZnO were fabricated. They exhibited enhanced antibacterial and mechanical properties. The experimental conditions allowed obtaining ZnO in the shape of rods and irregular sphere-like particles. However, no size/shape effect on antimicrobial response was detected. The boosting of the mechanical properties has been assigned mostly to chitosan; however, the tensile index was significantly improved in composites in comparison to chitosan-coated paper sheets. In addition, excellent antimicrobial activity was observed thanks to the co-application of chitosan and ZnO. Overall, the best antibacterial (100% of bacterial titer reduction) and mechanical properties (excluding tear index (MD)) were obtained for composites consisting of chitosan, bacterial cellulose and ZnO synthesized at 80 °C. Therefore, we assume that the designed paper coated with chitosan-bacterial cellulose-ZnO composites may be a promising eco-friendly composition in food packaging applications.

## Figures and Tables

**Table 1:** The effect of the different coatings on the mechanical properties of paper.

	Pristine Paper	Chitosan	ChBCsonTZnO	ChBCson80ZnO
Tensile index	a	b	c	d
[N m/g]	40.47 ± 0.35	43.20 ± 0.2	44.12 ± 0.21	44.52 ± 0.33
Tear index (MD *)	a	b	b	b
[mN m^2^/g]	5.66 ± 0.28	6.49 ± 0.28	6.44 ± 0.21	6.52 ± 0.26
Tear index (CD *)	a	b	c	c
[mN m^2^/g]	5.86 ± 0.28	7.32 ± 0.11	6.69 ± 0.36	6.77 ± 0.46
Burst index	a	bc	b	c
[kPa m^2^/g]	1.32 ± 0.08	1.55 ± 0.06	1.48 ± 0.04	1.60 ± 0.05

* MD: machine direction, * CD: cross direction, values marked with different letters (a, b, c, d) are significantly different (*p* < 0.05, ANOVA, Tukey test).

**Table 2:** The list of the tested samples.

Sample Abbreviation	Description of the Sample
P	Non-coated paper
Ch	Paper coated with chitosan solution
ChBCsonTZnO	Paper coated with chitosan solution with the addition of the BCNF modified with ZnO synthesized at room temperature
ChBCson80ZnO	Paper coated with chitosan solution with the addition of the BCNF modified with ZnO synthesized at 80 ℃

## Data Availability

Not applicable.
